# Identification of Accessible Hepatic Gene Signatures for Interindividual Variations in Nutrigenomic Response to Dietary Supplementation of Omega-3 Fatty Acids

**DOI:** 10.3390/cells10020467

**Published:** 2021-02-22

**Authors:** Yu Shi, Ping Li, Cheng-fei Jiang, Yi Chen, Yonghe Ma, Nikhil Gupta, Xiangbo Ruan, Haiming Cao

**Affiliations:** Cardiovascular Branch, National Heart, Lung and Blood Institute, National Institutes of Health, Bethesda, MD 20892, USA; shiyu0008@gmail.com (Y.S.); ping.li@nih.gov (P.L.); cheng-fei.jiang@nih.gov (C.-f.J.); yi.chen901@gmail.com (Y.C.); yonghe.ma@nih.gov (Y.M.); nikhil614@gmail.com (N.G.); xiangbo.ruan@nih.gov (X.R.)

**Keywords:** omega-3 fatty acids, cardiometabolic disease, hyperlipidemia, nonalcoholic fatty liver disease (NAFLD), nonalcoholic steatohepatitis (NASH), humanized mice, liver, exosome, biomarker

## Abstract

Dietary supplementation is a widely adapted strategy to maintain nutritional balance for improving health and preventing chronic diseases. Conflicting results in studies of similar design, however, suggest that there is substantial heterogenicity in individuals’ responses to nutrients, and personalized nutrition is required to achieve the maximum benefit of dietary supplementation. In recent years, nutrigenomics studies have been increasingly utilized to characterize the detailed genomic response to a specific nutrient, but it remains a daunting task to define the signatures responsible for interindividual variations to dietary supplements for tissues with limited accessibility. In this work, we used the hepatic response to omega-3 fatty acids as an example to probe such signatures. Through comprehensive analysis of nutrigenomic response to eicosapentaneoid acid (EPA) and/or docosahexaenoic acid (DHA) including both protein coding and long noncoding RNA (lncRNA) genes in human hepatocytes, we defined the EPA- and/or DHA-specific signature genes in hepatocytes. By analyzing gene expression variations in livers of healthy and relevant disease populations, we identified a set of protein coding and lncRNA signature genes whose responses to omega-3 fatty acid exhibit very high interindividual variabilities. The large variabilities of individual responses to omega-3 fatty acids were further validated in human hepatocytes from ten different donors. Finally, we profiled RNAs in exosomes isolated from the circulation of a liver-specific humanized mouse model, in which the humanized liver is the sole source of human RNAs, and confirmed the in vivo detectability of some signature genes, supporting their potential as biomarkers for nutrient response. Taken together, we have developed an efficient and practical procedure to identify nutrient-responsive gene signatures as well as accessible biomarkers for interindividual variations.

## 1. Introduction

The benefit of dietary/nutritional supplements (DS) in health management and disease prevention has long been recognized. Substantial evidence supports that nutritional prescriptions combined with adherence to other healthy lifestyles such as exercise could robustly reduce chronic disease burdens at the population level [1]. The consumption of dietary supplements in the United States has increased precipitously over recent decades. Currently, more than half of US adults and one-third of children use DS and the majority of these products contain essential nutrients. The most commonly consumed supplements are multivitamin/mineral products followed by calcium and omega-3 or fish oil supplements [2].

The wide adaptation of DS has created an urgent need for better understanding of how they impact metabolic health, and nutrigenomics has emerged as a novel and multidisciplinary research field to elucidate the interaction between food/nutrients and the human genome. One major direction of nutrigenomics is to comprehensively understand the genomic response to nutrients. Although great strides have been made in defining gene expression changes in response to nutrient supplements [3], several critical issues still need to be resolved before practical gene signatures for personalized medicine can be materialized. First, compared to nutrient-regulated genes, it is equally, if not more, important to identify those genes whose expressions reflect the interindividual variations but such studies have been currently lacking or limited. Mounting evidence supports that the effects of nutrition on health diverge significantly across or even within populations. For example, the supplement of omega-3 long-chain polyunsaturated fatty acids (*n*-3 PUFAs) has been shown to have beneficial effects, potentially by suppressing inflammation and improving lipid metabolism [4], but significant heterogeneity of such benefits has been observed. For instance, in terms of the plasma triglyceride (TG) lowering effect of *n*-3 PUFAs, 30% of the volunteers from the Fish Oil Intervention and Genotype (FINGEN) study were considered as non-responders and showed no reduction in TG after taking 1.8 g eicosapentaneoid acid (EPA) and docosahexaenoic acid (DHA) per day for 8 weeks [5]. Although the cardiometabolic benefits of *n*-3 PUFAs have been repeatedly demonstrated, they cannot be confirmed in recent meta-analyses with a very large cohort [6]. Obviously, conventional simplified dietary recommendations based on the average response of a population may not be sufficient and effective for all individuals. Identifying markers of interindividual variability in nutritional needs and responses is crucial for providing personalized advice to promote health and reduce chronic disease risk. Second, most gene expression analyses for nutritional effects have traditionally focused on protein coding genes. LncRNA genes, however, have become the dominant transcript class in the human genomes [7], and evidence is emerging that human lncRNAs play a critical role in the maintenance of metabolic homeostasis [8,9]. Particularly, lncRNAs are usually more tissue- and condition-specific than mRNAs [10], which could more specially reflect metabolic changes in key organs. Thus, it is anticipated that nutrient-responsive lncRNAs could serve as more robust biomarkers for individualized nutritional advice, but studies on lncRNAs in this area have clearly been lagging. Third, accessible gene signatures for internal metabolic organs need to be explored and defined. The majority of current studies have been focused on the gene expression changes in circulating blood cells due to them being easily accessible for sampling and marker detection. Nutrient-responsive gene signatures for primary metabolic organs in nutrient processing, particularly the liver, have yet to be established. This holds great promise for biomarker discovery if the transcripts of the nutrient-responsive genes from an internal metabolic organ can be detected in circulation or in extracellular vesicles such as exosome [11].

In this study, by analyzing protein coding and lncRNA genes, hepatic genes with high expression variabilities among both general and disease populations, and the RNAs detectable in exosome in the circulation of a liver humanized mouse model that permits the origin of human hepatocytes, we take the nutrigenomic response to omega-3 fatty acids in human hepatocytes as an example to establish a set of signature genes that could be responsible for or reflective of the interindividual variations in response to DS. 

## 2. Materials and Methods

### 2.1. RNA-seq Analysis Pipeline

RNA-seq data for primary human primary hepatocytes treated with omega-3 fatty acid have been deposited in NCBI Gene Expression Omnibus (GEO) (GSE165354). After quality control (FastQC: https://www.bioinformatics.babraham.ac.uk/projects/fastqc/ (accessed on 30 December 2020)) and trimming (TrimGalore: https://www.bioinformatics.babraham.ac.uk/projects/trim_galore/ (accessed on 30 December 2020)), fastq read files were aligned using HISAT2 to an index created using the GRCh38 genome. Aligned .sam files were then compressed into .bam files and sorted using Sambamba sort. Feature Counts from the subread package was used to generate the expression count level for each sample. Outlier samples with fewer than 1,000,000 reads aligned were removed. Protein-coding genes with CPM < 1 and lncRNA with CPM < 0.5 in fewer than half of the samples were excluded from further analysis.

### 2.2. NAFLD RNA Sequencing Data

The liver RNA-seq datasets for cross-sectional human studies of 192 non-alcoholic fatty liver disease (NAFLD) patients and 53 non disease patients were retrieved from BioProject PRJNA512027. RNA-seq cleaning, alignment, sorting, quantification, and filtering were conducted in the same way as primary hepatocyte RNA-seq analysis using this index and genome file. The same cutoff was applied as well.

### 2.3. Exosome-RNA Sequencing Data

RNA-seq data for humanized mice exosomes have been deposited in NCBI Gene Expression Omnibus (GEO) (GSE165350, access date (30 December 2020)). The exosomal RNA-seq reads were first mapped against a combined human and mouse genome with contigs for each annotation prefixed with “human_” and “mouse_”. Only the human-specific reads were used for further downstream analyses.

### 2.4. Differential Expression Analysis and PCA 

After combining technical replicates, the count data were subjected to the variance stabilizing transformation from the DESeq 2 package. Principal component analysis (PCA) was subsequently performed and the top two PCs were graphed to visualize clustering between experimental groups. Additionally, DESeq2 was used with non-normalized count data to find differentially expressed genes between experimental groups. Covariates were controlled for by adding them to experimental design if available. Cutoffs of |log2(FC)| > 0.2 and *p.adj* < 0.05 for protein coding genes or |Log2(FC)| > 0.15 and *p.adj* < 0.05 for non-coding genes were used for differential expression for all the human hepatocyte samples.

### 2.5. Gene Variability Analysis

#### 2.5.1. Population Expression Variability

To determine the hepatic gene expression variability in the healthy population, the human liver gene expression profile from GTEx v7 was employed. First, we applied an expression cut off of >1 CPM in 50% of samples to reduce mapped genes to 16,906 expressed genes including 2665 lncRNA genes in the liver. Thereafter, we quantified expression variability by calculating its coefficient of variation for each of the expressed genes across all the available samples. Similarly, the expression cut off of >1 CPM in half of the samples was applied for genes detected in NAFLD patient liver samples as mentioned above, and coefficient of variation was calculated accordingly.

#### 2.5.2. Hepatocyte Response Variability

Predicted highly variable signature genes responsive to omega-3 fatty acids were validated by qPCR. Their expression in response to DHA/EPA was normalized to those responding to vehicle treatment. These expression levels across the 10 donors were subsequently employed to calculate coefficient of variation as response variability.

### 2.6. Functional Gene Enrichment of Protein Coding Genes Responsive to DHA/EPA

DHA/EPA responsive protein coding genes (*p.adj* < 0.05) relevant in the NAFLD condition (i.e., overlapped with DEGs in NAFLD progression) were subjected to GO term analyses. Likewise, the DHA/EPA signature genes (*p.adj* < 0.05, |log2(FC)| > 0.2 but not responsive to OA) were functionally profiled with the representative enriched GO terms revealed in the dot plots. 

### 2.7. Functional Prediction of lncRNAs Using a lncRNA–mRNA Correlation Approach

To identify the potential function of the signature lncRNAs, pairwise Pearson correlations were calculated for each individual signature lncRNAs and hepatic protein coding genes in the general population. The correlation networks were constructed with either upregulated or downregulated signature lncRNAs and their correlated hepatic protein coding genes with |R > 0.6|, *p* value < 0.05. For those hepatic protein coding genes strongly correlated with specific signature lncRNAs, KEGG pathway analysis was performed and enrichment *p* values were adjusted using the Benjamini–Hochberg procedure.

### 2.8. Motif Finding and Enrichment Analysis

To identify mediators relaying the effect of omega-3 fatty acid regulating gene expression, we extracted sequences 2 kb upstream or downstream of the transcription start site (TSS) for all signature genes, intersected with DNase hypersensitivity peaks in hepatocytes and identified transcription factor binding motifs in CIS-BP by the MEME suite [12]. Thereafter, we performed motif enrichment analysis using the AME tool (Analysis of Motif Enrichment) from the MEME suite [13]. Randomly generated control sequences were created using AME’s shuffle function, from which enriched motifs for each group of signature genes have been identified. 

### 2.9. Primary Culture of Human Hepatocytes

The cryopreserved primary human hepatocytes from 10 donors were obtained from Thermo Fisher Scientific (Waltham, MA, USA), BioIVT (Westbury, NY, USA), and Lonza (Shady Grove, MD, USA). Manufacturers’ recommendations were followed when culturing primary human hepatocytes. Specifically, hepatocytes were thawed, pelleted, resuspended with plating medium (William’s medium E supplemented with Hepatocyte Plating Supplement Pack) (Thermo Fisher Scientific, Waltham, MA, USA), and seeded in a 24-well plate pre-coated with type I collage at a density of 5 × 10^5^ cells/well. For omega-3 fatty acid treatment, EPA, DHA, and OA from Cayman Chemical (Ann Arbor, MI, USA) were dissolved in ethanol and further diluted 1:1000 in incubation medium (serum free) containing 1% fatty acid free BSA (Sigma, St. Louis, MO, USA). Overnight plated primary human hepatocytes were cultured in serum-free incubation medium, washed, and then refed with incubation medium containing 1% fatty acid free BSA and different treatments (ethanol as vehicle or 200 µM DHA/EPA/OA) for 16 h before cells were harvested for RNA extraction. 

### 2.10. RNA Extraction, RNA-seq and Quantitative Real-Time PCR Analysis

Total RNA was isolated from primary human hepatocytes using a KingFisher PURE RNA tissue kit. The eluted RNA was subjected to strand-specific sequencing libraries construction with an Illumina TruSeq RNA sample prep kit and subsequently sequenced by NHLBI DNA sequencing and Genomic Core. Reverse transcription was performed with SuperScript^®^ III First-Strand Synthesis system (Invitrogen, Carlsbad, CA, USA) using 400 ng of RNA. Quantitative real-time RT-PCR was performed on a ViiATM 7 Real-Time PCR System (Applied Biosystems Inc, Beverly, MA, USA). The PCR program was: 2 min 30 s at 95 °C for enzyme activation, 40 cycles of 15 s at 95 °C, and 1 min at 60 °C. Melting curve analysis was performed to confirm the specific real-time PCR products. The full primer sequences used are provided below:


**Ensembl Gene ID**

**Gene Symbol**

**Sense Primer Sequence 5′-3′**

**Antisense Primer Sequence 5′-3′**

**ENSG00000072310**

*SREBF1*
GGAGCCATGGATTGCACTTT GGGTCAAATAGGCCAGGGAA 
**ENSG00000099194**

*SCD*
CAAGTGCCTCACCTCGAAAGTGTGTTCAGCAGGGTTTGTG
**ENSG00000110090**

*CPT1A*
GACAATACCTCGGAGCCTCACCACAGCATCAAGAGACTGC
**ENSG00000283568**

*18s*
AGTCCCTGCCCTTTGTACACACGATCCGAGGGCCTCACTA
**ENSG00000146592**

*CREB5*
AGGAAGAGGAGAGCAGCAAGAAGGTGCCTGAGTGATGACA
**ENSG00000131016**

*AKAP12*
GGTGGCGTACCTGACATAGAGGCTGAAGCACATCTTCTGG
**ENSG00000198431**

*TXNRD1*
GACCACGTTACTTGGGCATCGCACTCCAAAGCGACATAGG
**ENSG00000106366**

*SERPINE1*
CCGCCTCTTCCACAAATCAGGTAGGGCAGTTCCAGGATGT
**ENSG00000108984**

*MAP2K6*
TTCACAGAGACGTCAAGCCTTGGTTTGCAACCTGCATCAA
**ENSG00000137801**

*THBS1*
GCTCTACCAGTGTCCTCCTCTGGCTTGCAAGTCCTTTGTC
**ENSG00000054967**

*RELT*
TCTGGGTACTCATGGCTGTGCATGAGGCAGAAGACAGGGA
**ENSG00000147852**

*VLDLR*
TGCTCCGACCAATCTGATGAAGTTGACCTCATCACTGCCA
**ENSG00000114107**

*CEP70*
GGCTGAGGACACAGAGAAGATGATGCTACACAGCACCTGA
**ENSG00000088035**

*ALG6*
TGTGGCTTCCTTCGTTCTCTAATCCACGATCAACCGGGAA
**ENSG00000159082**

*SYNJ1*
CACCCAAGTTAGCTGGCATCACAGCCCAGAGCTTCTGATT
**ENSG00000119408**

*NEK6*
GATAGGCCGAGGACAGTTCAAGAGCCACTGTCTTCCTGTC
**ENSG00000131724**

*IL13RA1*
AGTCTGCTGTGACTGAGCTTGTGTCGGGACTGGTATTCCT
**ENSG00000066583**

*ISOC1*
AAATTCGTGGTGCAGCTGTTTTTCCCAGGGTAGTGAGCTG
**ENSG00000237767**

*LINC01370*
TGAGAGGCCATGTGGGTATGATCACAAGTCTGGCACCTCA
**ENSG00000248810**

*LINC02432*
GGGACAATGCAGGAACATGGATCTGGTGTCTGGGTCTTCC
**ENSG00000259953**

*AL138756.1*
TGGAGACACCAAACTCAGACACCAAGGGCACAAATCAGCTT
**ENSG00000172965**

*MIR4435-2HG*
CCTTCCATGCAAAGTTGGCTACACGCAGGAGTATCAGGAG

### 2.11. Exosomal RNA-Sequencing

HepaCur^TM^ serum from liver humanized FRG^®^KO mice was obtained from Yecuris (Tualatin, OR, USA) and subsequently sent to System Biosciences (Palo Alto, CA, USA) for Exo-NGS exosomal RNA-sequencing. Exosome isolation and subsequent RNA-sequencing were performed by System Biosciences. Specifically, exosomes were extracted from 500 μL liver humanized mice serum sample using their ExoQuick exosome isolation and RNA purification kits according to the manufacturer’s protocol (Cat # EQ806A-1).

## 3. Results

### 3.1. Nutrigenomic Response to Omega-3 Fatty Acid in Human Hepatocytes

In order to achieve precise nutrition based on individual response, the first step is to understand the holistic human nutritional response and the mechanisms by which nutrients affect human health [14]. Nutrigenomics is emerging to explore the effect of nutrients on the responsiveness of the human genome in multiple tissues/organs [15]. Although a plethora of studies have demonstrated the benefits of the dietary supplement (DS), omega-3 fatty acids (*n*-3 PUFAs), in reducing plasma TG levels, resolving inflammation, and improving insulin sensitivity [4,16], the genomic response to *n*-3 PUFAs has been mainly focused on blood cells which are readily accessible [17,18]. As the liver is the major organ for nutrient and lipid metabolism, we aimed to understand the hepatic nutrigenomic response to *n*-3 PUFAs.

**Figure 1:**
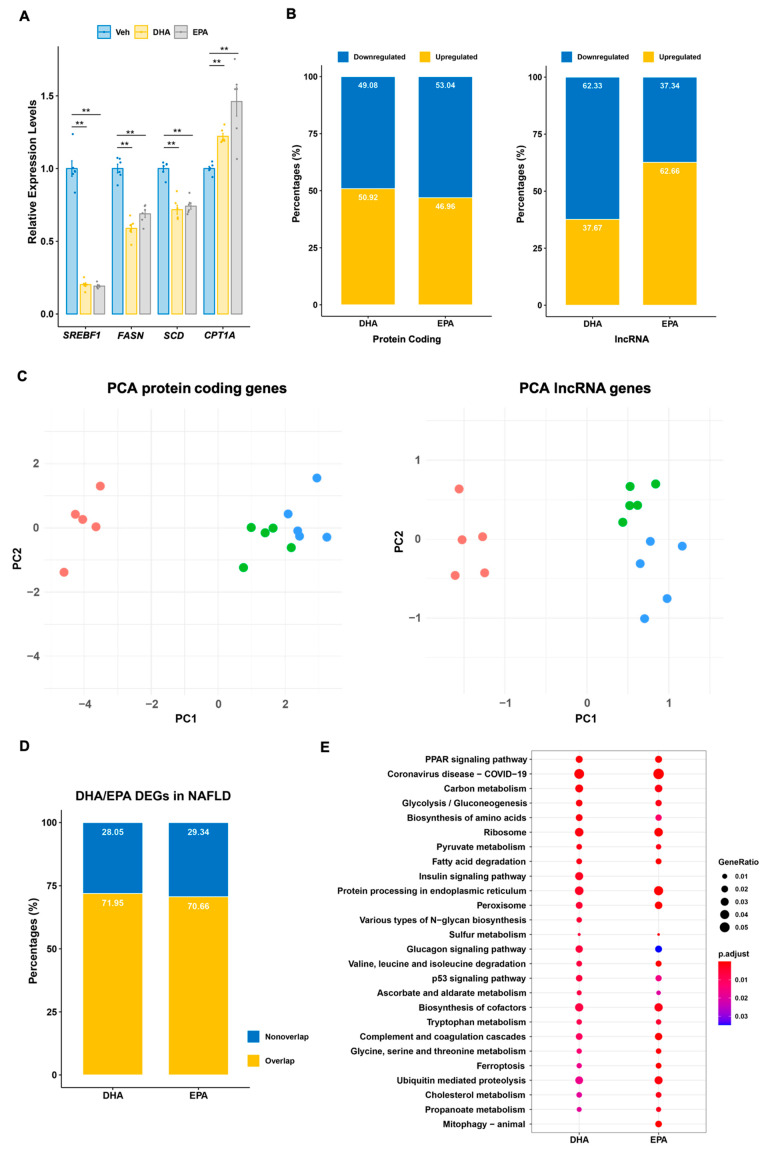
Nutrigenomic response to omega-3 fatty acid in human hepatocytes. (**A**) Expression of genes involved in de novo lipogenesis and fatty acid oxidation after 16h treatment with 200 µM DHA or EPA. Number of replicates = 5, error bar represents SEM, ** *p* < 0.001, two-tailed unpaired Student’s t-test. (**B**) Composition of differentially expressed genes responding to DHA or EPA. Bar charts representing the percentages of protein coding (left) or lncRNA (right) DEGs up or downregulated by DHA or EPA. (**C**) PCAs of expressed protein coding (left) and lncRNA (right) genes in primary hepatocytes in response to DHA/EPA/vehicle. (**D**) Relevance of *n*-3 PUFA DEGs in NAFLD. Percentage of *n*-3 PUFA responsive genes differentially expressed in NAFLD. (**E**) GO term analyses for *n*-3 PUFA responsive genes differentially expressed in NAFLD.

To that end, we treated human primary hepatocytes with EPA or DHA. The dose and duration (200 µM for 16 h) have been reported to effectively reduce the expression of genes involved in lipogenesis without eliciting significant cell death [19,20]. Consistent with published studies, we found that both treatments led to the suppression of lipogenic genes such as *SREBF1*, *FASN*, and *SCD*, and increased the expression of *CPT1A* indicative of enhanced fatty acid oxidation (Figure 1A). To unravel the genomic response to DHA or EPA in human hepatocytes, we performed transcriptomic profiling of the samples by strand-specific, pair-end deep RNA sequencing. Of 17,189 genes detected, we identified 4466 and 5415 genes significantly regulated by DHA or EPA compared to vehicle treatment, respectively (Supplementary Data 1 and 2). Among those regulated genes, 86.4% of DHA responsive and 84.4% of EPA responsive were protein coding genes; the remaining were non-coding within which lncRNA transcripts accounted for about 60%. The percentages of protein coding genes upregulated or downregulated were similar between DHA and EPA supplementation. Interestingly, unlike protein coding genes, the percentages of upregulated and downregulated lncRNAs differed strikingly between DHA and EPA. Specifically, about 2/3 of the DHA-responsive lncRNAs are downregulated as opposed to 1/3 of EPA-responsive ones (Figure 1B, Supplementary Data 3 and 4).

Despite the fact that both DHA and EPA exert beneficial functions such as lipid-lowering and anti-inflammation, a growing body of evidence has demonstrated these two *n*-3 PUFAs appear to have distinct functions [21,22,23,24]. To have an overview whether the different functions of these two *n*-3 PUFAs could be captured by transcriptomic profiling, principal component analyses (PCA) were performed on all regulated transcripts. As shown in Figure 1C, either protein coding or lncRNAs genes could readily separate all samples into distinct groups indicating that overall transcriptomic responses were different between DHA or EPA treatment.

As the liver is the central organ for metabolism in human, genes regulated by DS likely have implication in metabolic regulation. To determine the relevance of *n*-3 PUFA regulated genes observed in cultured hepatocytes in an in vivo pathophysiological process, we examined their regulation in human non-alcoholic fatty liver disease (NAFLD). NAFLD is a metabolic disorder of high prevalence and is known to cause global changes in gene expression and metabolism in the liver [25,26]. Specifically, we analyzed RNA-seq dataset composed of human liver samples from a cross-sectional study of NAFLD and found that the expression levels of ~72% of DHA-regulated and ~71% EPA-regulated genes were also changed in NAFLD, including 2769 protein coding genes and 199 lncRNA genes responding to DHA or 3228 protein coding genes and 255 lncRNAs responsive to EPA (Figure 1D, Supplementary Data 5 and 6). Functional enrichment analysis of regulated protein coding genes indicated they are related to crucial metabolic pathways (Figure 1E), supporting the notion that *n*-3 PUFA-regulated genes in hepatocytes may have direct involvement in the development of metabolic disease.

### 3.2. Protein Coding Signature Genes Responsive to DHA/EPA Supplementation 

**Figure 2:**
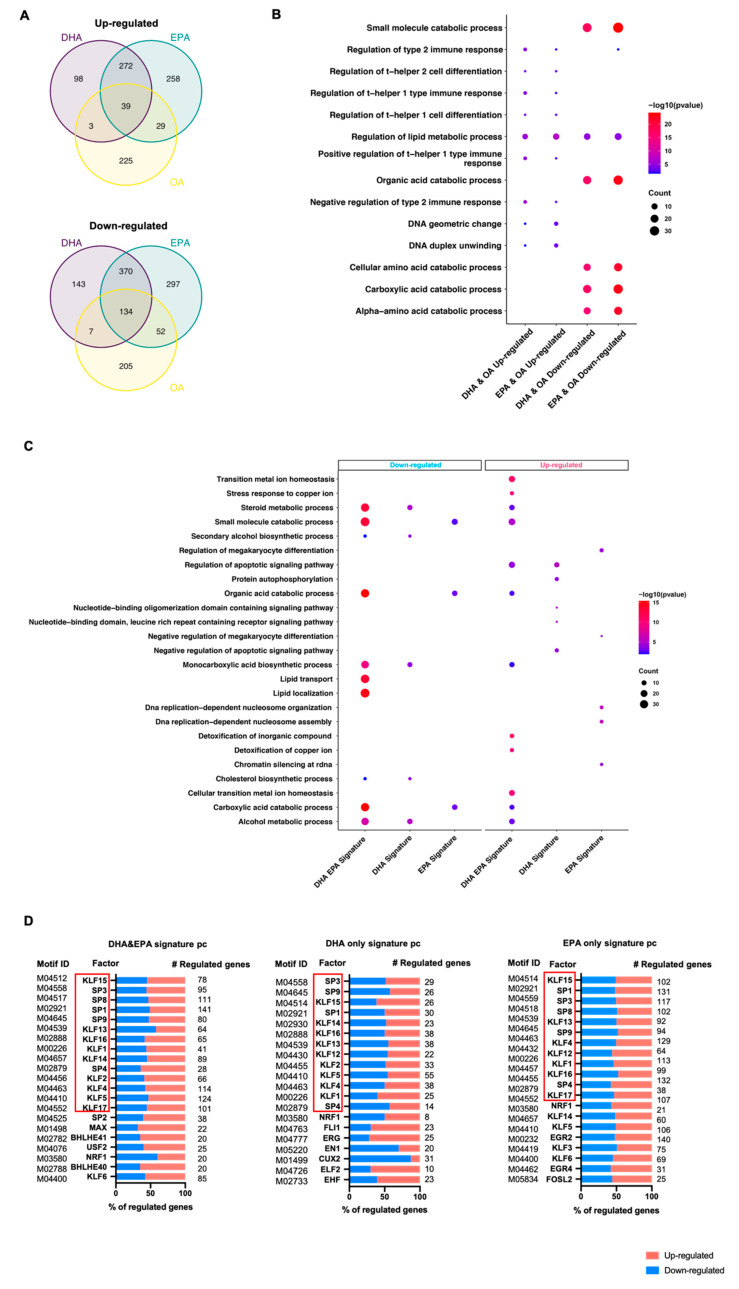
Identification of signature protein coding genes responsive to DHA/EPA supplementation. (**A**) Defining DHA and EPA signature protein coding genes by intersecting DEGs responsive to DHA, EPA, or OA. (**B**) GO Term analyses for DEGs coregulated by DHA and OA or EPA and OA. (**C**) Top 5 enriched GO terms for DHA and EPA commonly regulated, DHA-specific or EPA-specific signature protein coding genes. (**D**) Transcription factor binding motifs enriched in DHA, EPA-specific, or DHA and EPA commonly regulated signature protein coding genes.

Identification of gene signatures is not only important to understand the effect of n-3 PUFAs on hepatocytes, but also is a major step towards personalized nutrition by defining potential markers for a specific nutritional response. In order to define the signatures of n-3 PUFA supplementation, we included oleic acid treatment as a control to define the common effects of unsaturated fatty acids. We first focused on the protein coding genes, from which the regulated pathways could be directly inferred. By comparative analysis of protein coding genes which were respectively responsive to DHA, EPA, and Oleic acid (OA), 254 genes regulated by EPA (68 upregulated and 186 downregulated) or 183 genes responsive to DHA (42 upregulated and 141 downregulated) were found to be regulated by OA as well (Figure 2A, Supplementary Data 7 and 8). Those genes are mainly involved in the lipid and amino acid metabolism, immune responses, etc., which likely reflect the general effects of unsaturated fatty acids rather than the specific effects of n-3 PUFAs (Figure 2B). We were more interested in genes that were specifically responsive to DHA and/or EPA but not to oleic acid supplementation. Altogether, 241 protein coding genes responding to DHA only, 555 genes for EPA only, and 642 genes regulated by both DHA and EPA were identified (Figure 2A, Supplementary Data 9).

Pathway enrichment analyses of protein coding signature genes suggested DHA and EPA regulate a number of important metabolic pathways in human hepatocytes (Figure 2C). The most significantly affected processes are regulated by both DHA and EPA. Nearly half of these affected processes are shared by both upregulated and downregulated genes, including alcohol, steroid, and carboxylic acid metabolism pathways, etc. (Figure 2C). Interestingly, while DHA and EPA supplementation led to significant downregulation of signature genes involved in lipid transport and localization, the upregulated genes are mainly associated with processes related to metal ion homeostasis and alcohol metabolism.

Although most processes enriched in DHA- and EPA-regulated genes specifically are also affected by both DHA and EPA, we were able to reveal specific functions of DHA and EPA. For example, we identified upregulated protein autophosphorylation and negative regulation of apoptotic signaling pathways in response to DHA, and DNA replication related nucleosome assembly and organization in response to EPA (Figure 2C). 

To further understand how the signature genes, both up and downregulated, were regulated by DHA and/or EPA, we attempted to identify the regulatory elements on these genes and potential corresponding binding transcription factors (Figure 2D). The promoter flanking sequences on these regulated genes with high DNA accessibility defined by the DNase I hypersensitive sites in human liver were analyzed for enriched motifs. Strikingly, the binding sites for the Krüppel-like factor and specificity protein family (KLF/SP) and NRF1 were highly enriched in EPA- and/or DHA-regulated genes. Sites for certain BHLHE factors were also enriched in EPA and DHA commonly regulated signature genes. 

Interestingly, fatty acid specific sites were also identified. For example, FLI1 and CUX2 sites were found for only DHA signature genes, while EGR2/4 and FOSL2 sites were found for only EPA signature genes. Moreover, promoter regions of EPA/DHA regulated genes were enriched for novel motifs with no known binding TFs, which might also mediate the hepatic gene expression in response to *n*-3 PUFA supplementation (Supplementary Figure S1A).

### 3.3. Signature lncRNA Genes Responsive to DHA/EPA Supplementation

**Figure 3:**
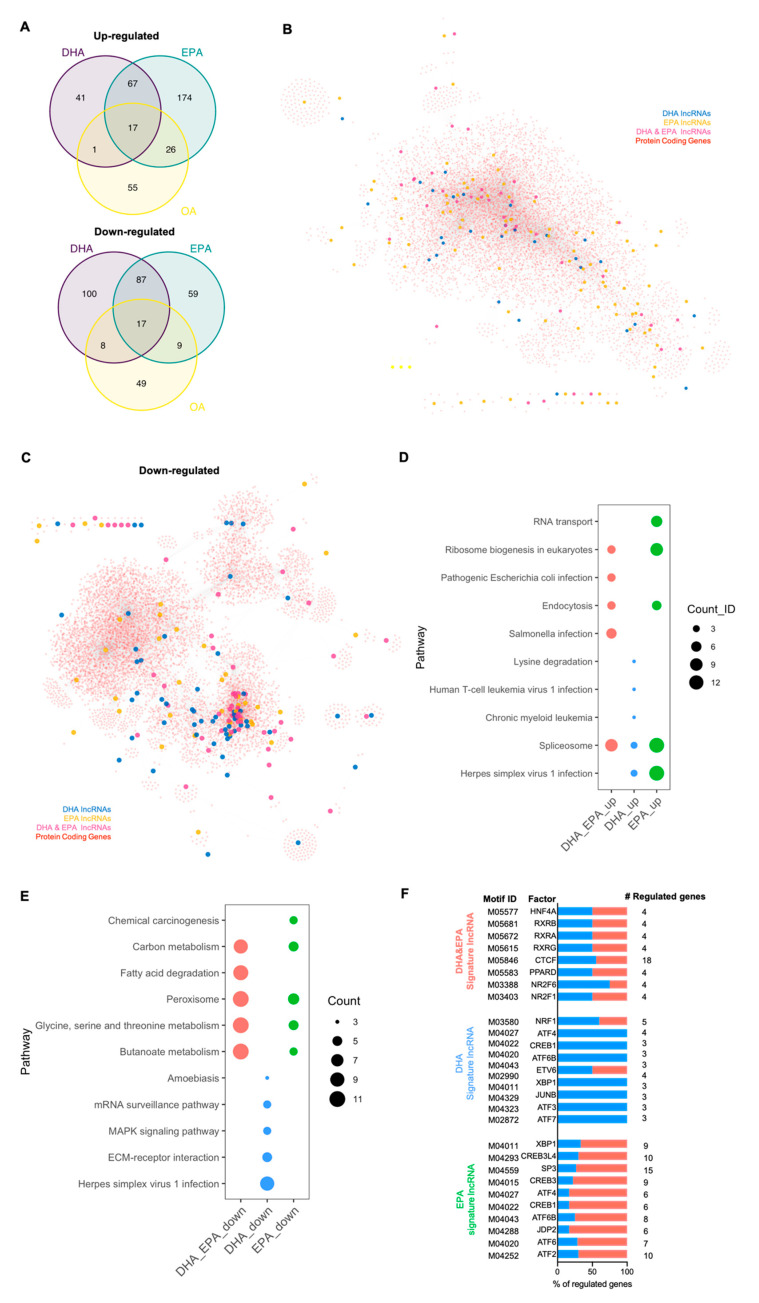
Define signature lncRNA genes responsive to DHA/EPA supplementation. (**A**) Define signature lncRNA genes by intersection of DHA, EPA, or OA responsive lncRNAs. (**B**,**C**) lncRNA-mRNA co-expression network analysis to infer the potential functions of signature lncRNA genes. Networks were constructed with upregulated (**B**) or downregulated (**C**) signature lncRNAs and their strongly correlated hepatic protein coding genes (Pearson cor |R| > 0.6, *p* value < 0.05). (**D**,**E**) Top KEGG pathways mostly enriched by each category of upregulated (**D**) and downregulated (**E**) signature lncRNAs. Specifically, pathway enrichment analysis was performed for each signature lncRNA based on their correlated protein coding genes. The resultant pathways with enrichment score (*p* value < 0.05) were subsequently counted to calculate the top 5 most enriched representative pathways. (**F**) Transcription factor binding motifs enriched in DHA, EPA-specific or DHA and EPA commonly regulated signature lncRNA genes.

Since lncRNAs accounted for the majority of the noncoding DEGs (Figure 1B), we also identified signature lncRNA genes in parallel representing the impact of DHA/EPA on noncoding transcriptome. The number of signature lncRNA genes identified is 141, 233, and 154 for DHA-only, EPA-only, and DHA and EPA in common, respectively (Figure 3A, Supplementary Data 10). As aforementioned, the relative numbers of upregulated and downregulated lncRNAs responsive to DHA or EPA supplementation were quite distinct (Figure 1C). Specifically, for signature lncRNAs, the number of downregulated signature lncRNAs responding to DHA only is more than two-fold of those upregulated (41 upregulated vs. 100 downregulated). EPA, on the contrary, mainly exerted its function via inducing rather than suppressing lncRNA expression (174 upregulated vs. 59 downregulated). For signature lncRNAs co-regulated by DHA and EPA, the number of upregulated lncRNAs was quite close to those downregulated (67 upregulated vs. 87 downregulated). Therefore, signature lncRNAs may play potentially more important roles attributing to the distinct functions of EPA and DHA and also may serve as more specific signatures for distinct *n*-3 PUFAs.

Recent works, including ours, have demonstrated that lncRNAs play important roles in metabolism regulation [8,9,27,28]. To explore the potential function of these signature lncRNAs, we performed lncRNA–mRNA correlation analysis between each individual signature lncRNA and protein coding gene in human liver (GTEx dataset). Networks of lncRNA and their highly correlated protein coding genes were constructed to reveal their association. Despite some clustering, the distribution of majority hub lncRNAs is quite distinct, suggesting shared and specific functions among each group of signature lncRNAs (Figure 3B,C). To further infer the function of a specific group of signature lncRNAs, pathway enrichment analysis was performed based on highly correlated hepatic coding genes and the top 5 most enriched pathways for each group of signature lncRNAs were calculated accordingly. As shown in Figure 3D, for upregulated signature lncRNAs, either DHA and EPA coregulated, DHA or EPA specifically regulated lncRNAs were predicted to have potential roles in RNA splicing, ribosome biogenesis, and endocytosis. Interestingly, DHA- and EPA-regulated lncRNAs could be related to pathogen infections, with DHA and EPA coregulated lncRNAs involved in bacterial infection response, while DHA and/or EPA specific lncRNAs, instead, are involved in viral infection response. For downregulated signature lncRNAs, the potential functions are similar between DHA and EPA coregulated and EPA specifically regulated lncRNAs including pathways related to peroxisome and amino acid metabolism, while DHA-regulated lncRNA has some unique functions such as MAPK signaling and ECM–receptor interaction (Figure 3E). 

As with protein coding genes, promoter flanking sequence analysis revealed enriched motifs for the signature lncRNA genes (Figure 3F and Supplementary Figure S1B). For example, sites for CREB and ATF families as well as XBP1 were enriched in either EPA- or DHA-regulated lncRNA genes, while sites for the RXR family were enriched for lncRNA genes regulated by both EPA and DHA. Interestingly, the enriched sites were mostly found in EPA specifically upregulated lncRNA genes, while they were mostly found in DHA specifically downregulated ones. For the lncRNA genes regulated by both DHA and EPA, the enriched sites were evenly distributed in upregulated or downregulated ones.

### 3.4. Biomarker Genes Contributing to Individual Variation in Nutritional Response to DHA/EPA 

Many studies have demonstrated that the majority of responsive genes, regardless of the nature of the stimuli, were uniquely regulated among individuals [29,30]. Intuitively, those inducible genes exhibiting heterogeneous responses are more likely contributing to the variable phenotypes observed. Within the DHA/EPA signature genes identified, we then set off to select genes exhibiting diverse responses among individuals thereby contributing to the interindividual variation in nutritional response to DHA/EPA. Those types of signature genes could potentially serve as predictive biomarkers for the responsiveness of each individual to DHA/EPA. 

**Figure 4:**
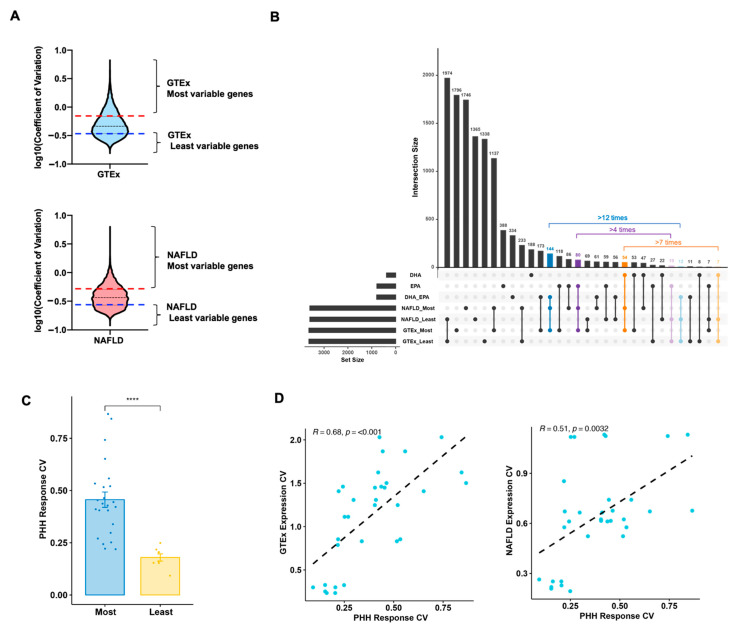
Selection of biomarker genes contributing to individual variation in hepatic response to DHA/EPA. (**A**) Defining genes with most or least expression variability in general (GTEx) or relevant disease (NAFLD) populations. (**B**) Selection of signature genes displaying high population expression variability in general (GTEx) and relevant metabolic disease (NAFLD) populations, while signature genes with least population expression variability in human populations served as negative controls. Here, the UpSet plot summarized the intersections of each group of signature genes and the most or least variable hepatic genes in general/relevant disease populations. (**C**) Response coefficient of variation (CV) for signature genes with high expression variability was significantly higher than those exhibiting low expression variability in human populations. Coefficient of variation was calculated to represent the response variability based on their responses across a panel of 10 donors. **** *p* < 0.001, two-tailed unpaired Student’s t-test was performed to compare most variable versus least variable signature genes. (**D**) Correlation of coefficient of variation between the expression in general/relevant disease populations and responses in PHH (Primary human hepatocytes) were plotted.

We reason that genes with higher expression variability across individuals within a population would exhibit larger individual variations in response to stimuli. To investigate this postulate, we first evaluated the variability of hepatic expression for DHA/EPA signature genes in both the general (GTEx) and metabolic disease relevant (NAFLD) populations. This was performed by intersecting the DHA/EPA signature genes (both protein coding and lncRNA genes) with the most variable (top quantile) or least variable (bottom quantile) hepatic genes in general and NAFLD populations (Figure 4A, Supplementary Data 11 and 12). The number of most variable signature genes co-regulated by both DHA and EPA was about 12 times more than those exhibiting the least variability. For DHA only and EPA only signature genes, these ratios were also as high as about 7 and 4 times (Figure 4B), respectively. Together, the expression of the majority of DHA/EPA signature genes was highly variable among individuals. 

Thereafter, the responsiveness to *n*-3 PUFA of these most variable signature genes in the human population was further studied in primary hepatocytes from a panel of 10 independent donors. Those tested signature genes with high population expression variabilities also exhibited high response variability across the panel of donors which is significantly higher than those with least expression variability (Figure 4C). More importantly, the expression coefficient of variation for signature genes, either in general (GTEx) or disease (NAFLD) populations, was well correlated with the response coefficient of variation in primary hepatocytes (Figure 4D). Therefore, the heterogeneous transcriptomic responses were successfully predicted by the expression variability in humans. Those most variable DHA/EPA signature genes represent a list of candidates which could be employed as potential biomarkers for individual variation of response to DHA/EPA in humans. 

### 3.5. Detection of Transcripts of DHA/EPA Biomarker Genes in Liver-Derived Exosomes in Circulation 

Biomarkers have been widely implicated in the diagnosis and management of a wide range of diseases including cancer, cardiovascular disease, infection, and genetic disorders [31]. However, robust and reproducible nutritional biomarkers are still very limited, requesting more efforts for novel biomarker discoveries [32]. Here, in the current study, we would like to explore the possibilities of the most variable signature genes as potential biomarkers representing the hepatic transcriptomic responses to DHA/EPA. 

Exosome, the membraned vesicles containing a wide variety of DNA, RNA, proteins, and lipids representing the molecular composition of their parent cells, has been increasingly recognized as a rich source for biomarker discovery [11]. Hence, it is tempting to examine whether the most variable signature genes, either protein coding or lncRNA genes, could be detected in liver-derived exosomes.

**Figure 5:**
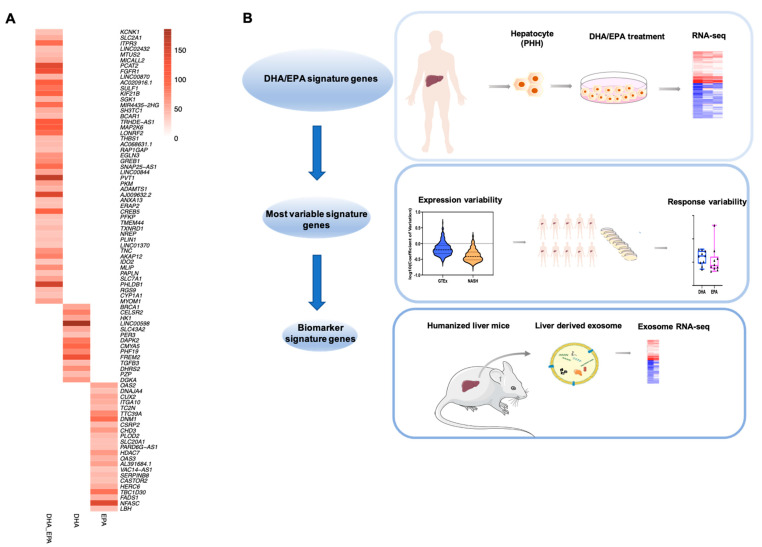
Detection of transcripts for DHA/EPA biomarker genes in liver-derived exosomes in circulation. (**A**) Detection of biomarker signature genes in humanized liver-derived exosomes by intersection of the top 20% human genes with the highest expression level (based on count value) in humanized liver-derived exosome with highly variable signature genes. (**B**) A road map defines biomarker genes representing interindividual variations of hepatic responses to DHA/EPA.

Exosomes obtained from human circulation could be derived from a variety of tissues and organs [11,33,34]. To obtain exosomal RNA samples specifically originating from human liver in an in vivo setting, we employed a liver-specific humanized mouse model which has been previously demonstrated by our group to be suitable for studying the regulation of human hepatic genes under well-controlled experimental conditions [8]. For transcripts of human genes that are readily detectable in the exosomes from the circulation of the mouse with the humanized liver, the origin of the human hepatocytes can be determined for certain. The plasma from the humanized mice was used for isolation of exosomal RNAs, which were then subjected to RNA-seq analysis. The top 20% human genes with the highest detectable level in exosomes (Supplementary Data 13), representing the best candidates for potential biomarkers, were examined for the existence of the most variable signature genes described above. As shown in Figure 5A, we identified transcripts in circulation from the 49 most variable signature genes responding to DHA and EPA, 14 responsive to DHA only, and 23 for EPA only (Supplementary Data 13). These circulating transcripts, corresponding to most variable genes, represent the promising candidate markers for the hepatic responses to DHA and EPA with the largest interindividual variability. 

Thus, as shown in Figure 5B, by analyzing nutrigenomic response, testing gene expression variability both in human populations and in nutrient-treated cells from different donors, and examining RNAs in liver-specific derived exosomes in vivo in a humanized mouse, we have developed a pipeline to define the signature genes which are potentially responsible for the individual variations in nutritional response.

## 4. Discussion

For the most effective and safe management of diseases in an individualized manner, precision medicine has started to be practiced by health care providers, particularly for cancer treatment. For better improving our health and preventing diseases, it has also been realized that dietary/nutritional recommendations should be tailored to individual needs and responses. Recently, NIH released a strategic plan to accelerate nutrition research over the next 10 years with a focus on precision nutrition. One of the major efforts that should be undertaken is to define biomarkers reflecting interindividual variations in nutritional responses. With this aim, usually a large number of samplings and unbiased measurements are required. In this work, we have explored a practical and cost-effective approach to search for such nutritional responsive biomarker genes by combinatory analysis of nutrigenomic response in an in vitro model as well as gene expression variability among human populations of a relevant tissue, the hepatic response to omega-3 fatty acids in our case. Moreover, specific detection in circulation exosomes suggests their promising potential as an accessible surrogate for the responsiveness of a conventionally inaccessible internal organ.

Our work presents a robust and innovative approach to determine gene signatures reflecting the variabilities in response to DS. In order to define the transcriptional responses that differ among individuals, sampling from a large cohort followed by transcriptome analysis is the standard approach, which is expensive yet not efficient. Sometimes, it is even impracticable to carry out such studies with internal organs such as the liver. Our approach, instead, harnesses the intrinsic expression variability of the genes across human individuals to identify the potential transcriptomic signatures that reflect variable responses to DS. Indeed, among the genes responsive to omega-3 fatty acids in hepatocytes, those with the most variable expression level among humans were confirmed to show much higher variations in response to omega-3 fatty acids than those genes with the least variability. Presumably, the fold changes of omega-3 fatty acids-responsive genes contributing to interindividual variation should be heterogenous among different donors. We loosen the initial fold change cutoff to capture as many as possible of the responsive genes. Moreover, although the omega-3 fatty acids treatment was performed in cultured cells, our approach to cross reference in vivo and in vitro data can potentially limit the cell culture artifacts to a certain extent. 

A key value and uniqueness of our gene signature is its accessibility, particularly for the liver whose metabolic signatures in healthy individuals are otherwise very difficult to ascertain. Since DS is usually not intended to treat severe or urgent diseases, for biomarkers reflecting the nutritional response, easy accessibility is particularly important. The detectability of the RNAs in circulating exosomes from the liver-specific humanized mice is an intriguing finding. In a real clinical setting, however, it is still challenging to determine the origins of RNAs in exosomes isolated from blood. Our work addressed this question by extending our analysis to lncRNAs whose expression is much more tissue-specific than mRNAs. For example, one of the validated most variable lncRNA detected in humanized liver-derived exosome, linc01370, is specifically expressed in liver and it is a strong candidate biomarker for clinical testing. 

While we are taking hepatic nutrigenomic response to *n*-3 PUFAs as an example to explore the interindividual variations, we certainly gain better understanding regarding the effects of DHA and EPA in human hepatocytes. Specifically, in addition to protein coding genes, we have also identified a substantial number of DHA- and EPA-regulated lncRNAs in hepatocytes, which we found to be more specific than protein-coding genes to distinguish the effect of different *n*-3 PUFAs. Meanwhile, lncRNA-mRNA co-expression analysis using large human liver transcriptome datasets such as GTEx allowed us to predict the functions of these lncRNAs. The potential involvement of lncRNAs in metabolism and other important cellular pathways makes it worthwhile to further investigate the roles of lncRNAs in the regulation of nutritional response. Corroborating with the DHA- or EPA-regulated genes, common and specific regulatory elements are found on their regulated genes. Interestingly, high enrichment of binding sites of the KLF/SP family was found on their regulated coding genes, while the RXR family was found on lncRNA genes. Both of these factors are important families of transcript factors involved in diverse functions in liver [35,36]. Further study of these regulators in mediating the effects of DHA/EPA in liver would improve our understanding of the hepatic response to omega-3 fatty acids. 

Thus, starting with the comprehensive analysis of the genomic response to omega-3 fatty acids in human hepatocyte from a single donor, we confirmed the highly variable responses of selected genes among hepatocytes from multiple donors. With increasing attention on precision nutrition, strategies defining the interindividual variations in nutritional responses in additional cell/tissue types would become more critical and urgent, and we demonstrated in this work the possibility of a cost-effective approach to define such signatures. 

## Data Availability

Data are contained within the article or Supplementary Material.

## Supplementary Materials

The following are available online at https://www.mdpi.com/2073-4409/10/2/467/s1, Figure S1. Regulatory element analysis of signature genes defines novel motifs A. Top 10 novel motifs enriched for DHA and EPA commonly, DHA or EPA specifically regulated signature protein coding genes. B. Top 5 novel motifs enriched for signature lncRNA genes, Supplementary Data 1: DEGs responsive to DHA treatment in primary human hepatocytes, Supplementary Data 2: DEGs responsive to EPA treatment in primary human hepatocytes, Supplementary Data 3: lncRNA genes up or down-regulated by DHA in primary human hepatocytes, Supplementary Data 4: lncRNA genes up or down-regulated by EPA in primary human hepatocytes, Supplementary Data 5: DHA regulated DEGs relevant in NAFLD progression, Supplementary Data 6: EPA regulated DEGs relevant in NAFLD progression, Supplementary Data 7: DHA regulated DEGs coregulated by OA, Supplementary Data 8: EPA regulated DEGs coregulated by OA, Supplementary Data 9: Protein coding signature genes responsive to DHA&EPA, DHA only or EPA only, Supplementary Data 10: lncRNA signature genes responsive to DHA&EPA, DHA only or EPA only, Supplementary Data 11: Most variable signature genes in general (GTEx) and disease relevant (NAFLD) populations, Supplementary Data 12: Least variable signature genes in general (GTEx) and disease relevant (NAFLD) populations, Supplementary Data 13: Biomarker signature genes detected in humanized liver-derived exosomes.
