# Supplementary material for: Identification of Accessible Hepatic Gene Signatures for Interindividual Variations in Nutrigenomic Response to Dietary Supplementation of Omega-3 Fatty Acids
Source: Cells. 2021 Feb 22;10(2):467. doi: 10.3390/cells10020467 (PMC7926558; doi:10.3390/cells10020467)
Supplement: Supplementary file 1 [file cells-10-00467-s001.zip › cells-1094174-resubmitted supplementary/Supplementary data/Supplementary Figure .pdf]

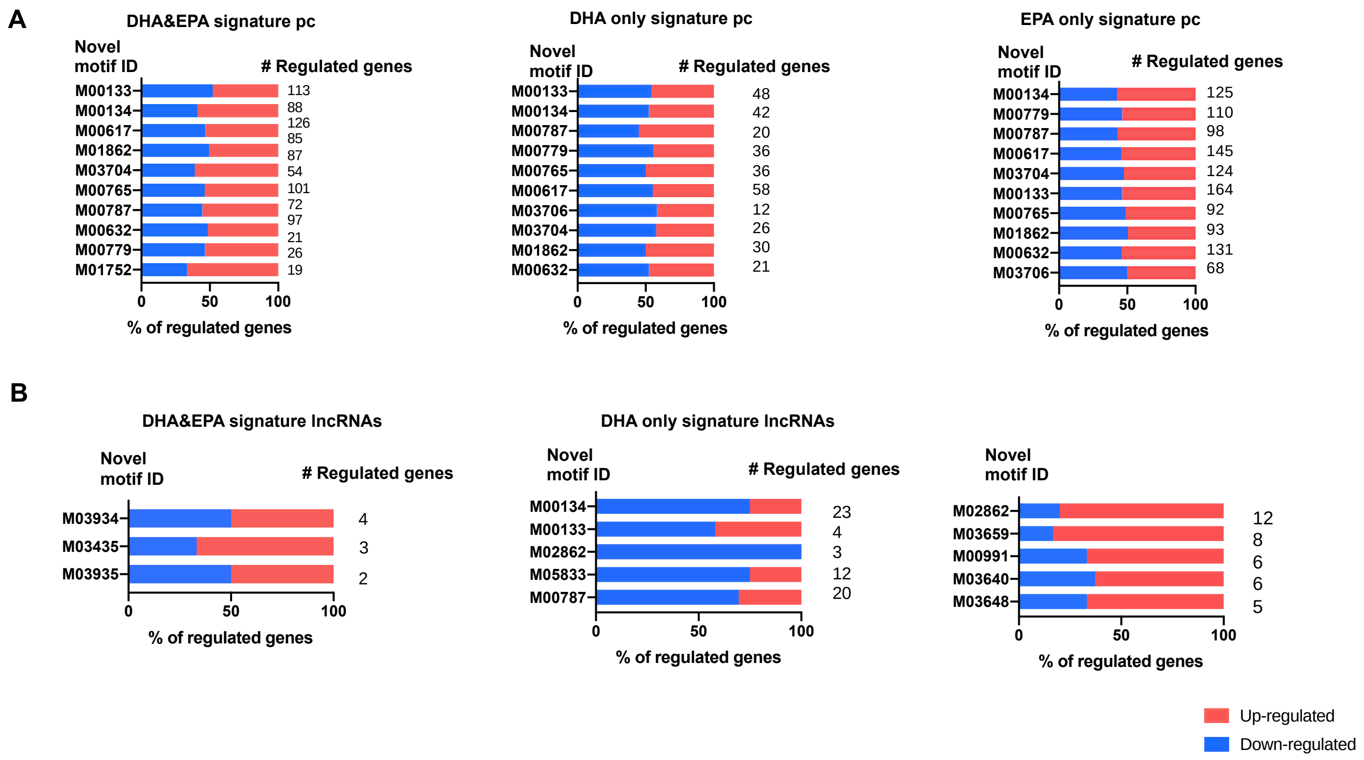

Supplementary Figure 1. Regulatory element analysis of signature genes defines novel motifs. (A) Novel motifs enriched in signature protein coding genes. Top 10 novel motifs enriched for DHA and EPA commonly and DHA or EPA specifically regulated signature protein coding genes. (B) Novel motif enriched in signature lncRNA genes. Top 5 novel motifs enriched in signature lncRNA genes.
